# Dietary antioxidants and flavonoids are inversely associated with prostate cancer risk and mortality: evidence from NHANES and machine learning

**DOI:** 10.3389/fnut.2025.1611848

**Published:** 2025-07-08

**Authors:** Yanyang Jin, Dong-shan Lv, Li-po Zhou, Li Xiao, Guang-quan Tong, Abdallah Karim, Shuai Xue, Hongyang Tian, Cheng-cai Wang, Kun Feng, Ding-ming Song, You-liang Guan

**Affiliations:** ^1^Department of Urology, The First Affiliated Hospital of Jinzhou Medical University, Jinzhou Medical University, Jinzhou, Liaoning, China; ^2^Department of Obstetrics and Gynecology, Jingzhou Hospital, Yangtze University, Jingzhou, Hubei, China

**Keywords:** prostate cancer, antioxidants, flavonoids, selenium, magnesium, NHANES, machine learning

## Abstract

**Background:**

Oxidative stress and dietary micronutrient imbalances have been implicated in prostate cancer (PCa) development and progression. Although flavonoids and antioxidants show promise in experimental models, evidence from population-based studies remains limited.

**Objectives:**

This research aimed to investigate the relationship between the consumption of antioxidants and flavonoids in the diet and the risk and survival of PCa, as well as to assess the potential of machine learning models in identifying significant dietary factors.

**Methods:**

Data from 2,629 male participants aged ≥40 years from National Health and Nutrition Examination Survey (NHANES) 2007–2010 were analyzed. Dietary intake was estimated using two 24-h recalls linked to the USDA Flavonoid Database. PCa status was self-reported. Survey-weighted logistic regression and Cox models evaluated associations with PCa prevalence and all-cause mortality, adjusting for demographic, lifestyle, and clinical covariates. Nine supervised machine learning models, including random forest (RF), were developed and validated. Shapley Additive Explanations (SHAP) values identified key predictors and visualized their effects.

**Results:**

Among 2,629 U.S. male participants from NHANES 2007–2010, 144 reported a history of PCa. Compared with non-cancer individuals, cases had lower intake of selenium, magnesium, quercetin, kaempferol, epicatechin, epigallocatechin, total flavones, and total flavonoids (all *P* < 0.05). Higher intake of selenium, magnesium, catechin, and myricetin was associated with reduced PCa risk in weighted regression models, with selenium remaining significant after multivariable adjustment [odds ratio (OR) = 0.50, 95% confidence interval (CI): 0.33–0.76]. Lower intake of selenium, magnesium, luteolin, quercetin, kaempferol, and total flavones was linked to increased mortality risk, and selenium independently predicted improved survival [hazard ratio (HR) = 0.69, 95% CI: 0.54–0.88]. The RF model showed superior predictive performance [area under the curve (AUC) = 0.740], identifying selenium, luteolin, total flavones, myricetin, catechin, and magnesium as key features. SHAP analysis revealed U-shaped associations for selenium, catechin, and myricetin, and dose-dependent protective effects for luteolin and magnesium.

**Conclusion:**

Our results highlight selenium, magnesium, and select flavonoids as promising dietary factors in reducing PCa risk and improving prognosis. These insights support the development of evidence-based, individualized nutritional strategies and call for further mechanistic and clinical investigations.

## 1 Introduction

Prostate cancer (PCa) ranks as one of the most prevalent cancers in men, with more than 1.4 million new cases globally in 2020. This corresponds to an age-standardized incidence rate (ASR) of 31 per 100,000 and an estimated lifetime risk of about 3.9%, positioning it as the second most commonly diagnosed cancer among men (1). Although factors such as age, ethnicity, and family history are well-recognized as significant risk factors for PCa, emerging research suggests that modifiable lifestyle choices and dietary habits may play a crucial role in improving overall survival, especially by reducing cardiovascular and all-cause mortality in individuals with PCa (2).

Oxidative stress contributes significantly to prostate carcinogenesis by inducing deoxyribonucleic acid (DNA) and mitochondrial damage, promoting chronic inflammation, and disrupting key signaling cascades within the tumor microenvironment (3, 4). Natural antioxidants, have been shown to exert protective effects against cancer development by neutralizing reactive oxygen species (ROS), regulating cell signaling pathways, and promoting apoptosis. Micronutrients like selenium, vitamin C, vitamin E, and magnesium, have demonstrated antioxidant and anti-inflammatory properties, contributing to reduced proliferation and enhanced apoptosis in various cancer cell types, including PCa (5).

Beyond conventional antioxidants, dietary flavonoids—a diverse group of polyphenolic compounds abundant in fruits, vegetables, teas, and legumes—have attracted increasing attention for their chemo-preventive roles in PCa. Experimental studies have demonstrated that flavonoids can regulate multiple cellular processes including oxidative stress, inflammation, cell proliferation, apoptosis, and epigenetic modulation (6). Specific subclasses, such as flavones (e.g., apigenin and luteolin), flavonols (e.g., quercetin and kaempferol), flavanones (e.g., naringenin), and isoflavones (e.g., genistein and daidzein), have shown the ability to inhibit tumor growth, modulate androgen receptor (AR) signaling, and reverse aberrant DNA methylation and histone modification in PCa models (7–10). These compounds can also affect non-coding ribonucleic acids (RNAs) such as microRNAs and lncRNAs, further influencing gene expression and cancer progression. Despite promising mechanistic evidence, large-scale epidemiological studies remain limited and findings are often inconsistent, underscoring the need for further population-based investigations to clarify the protective role of flavonoids in PCa (6).

Traditional statistical approaches may be limited in detecting complex, non-linear interactions between dietary components and disease outcomes. In contrast, machine learning algorithms, such as random forest (RF), offer enhanced flexibility in modeling high-dimensional and heterogeneous data, enabling more accurate risk prediction (11). In this context, the integration of Shapley Additive Explanation (SHAP) values further improves model interpretability by quantifying the contribution of each dietary feature to individual predictions (12). This approach not only strengthens predictive performance but also provides complementary insights into key nutritional determinants of PCa risk beyond those identified by conventional regression analyses.

This research sought to examine the relationship between the intake of dietary antioxidants and flavonoids and the prevalence and survival of PCa in United States (U.S.) adult males using National Health and Nutrition Examination Survey (NHANES) 2007–2010 data. Additionally, machine learning approaches were utilized to assess predictive accuracy and identify the most significant dietary factors. The results of this study could enhance our understanding of the dietary components linked to PCa and provide insights for developing future nutritional approaches for cancer prevention and treatment.

## 2 Materials and methods

### 2.1 Data source and study population

Data for this study were sourced from the 2007–2008 and 2009–2010 cycles of the NHANES, which utilizes a multistage, stratified probability sampling method to gather health and nutrition data representative of the civilian, non-institutionalized U.S. population. We focused on male participants aged 40 years and older who had complete information on PCa status, dietary intake of antioxidants and flavonoids, and relevant covariates. Individuals with incomplete demographic or dietary data were excluded from the analysis. Following the application of exclusion criteria, a total of 2,629 male participants remained for the final analysis.

### 2.2 Prostate cancer assessment

Prostate cancer status was classified according to self-reported physician diagnoses obtained from the NHANES medical conditions questionnaire. Participants who answered “Yes” to the question, “Has a doctor or other health professional ever informed you that you had prostate cancer?” were classified as PCa cases, while those who responded “No” were considered controls.

### 2.3 Dietary intake of antioxidants and flavonoids

Dietary intake data were obtained from two 24-h dietary recall interviews conducted in the NHANES, typically separated by 3–10 days. The average daily intake was calculated by taking the mean of the day 1 and day 2 dietary recall data. Antioxidants analyzed included vitamin A, vitamin C, vitamin E (alpha-tocopherol), carotene, magnesium, zinc, and selenium. A comprehensive flavonoid profile was constructed by linking NHANES dietary data to the United States Department of Agriculture (USDA) Expanded Flavonoid Database, available through the Food and Nutrient Database for Dietary Studies (FNDDS) website.^1^ Flavonoid subclasses included anthocyanidins, flavonols, flavones, flavanones, and flavan-3-ols. Total flavonoid and anthocyanidin intake were also calculated. All flavonoid intake estimates were derived as the mean of the two 24-h dietary recalls.

### 2.4 Covariates

The covariates considered in this study included sociodemographic, lifestyle, and clinical characteristics. Sociodemographic factors included age, race/ethnicity (non-Hispanic White, non-Hispanic Black, Mexican American, other Hispanic, and other races, including multi-racial), education level (≥high school vs. <high school), marital status (married/living with a partner vs. living alone), and the poverty income ratio (PIR), which was categorized as <1.3, 1.3–3.5, and ≥3.5. Lifestyle factors encompassed smoking status (never, former, and current) and alcohol consumption, which was classified as non-drinker/light drinker (including never, former, and mild consumption of ≤2 drinks/day) or heavy drinker (>2 drinks/day).

Clinical characteristics included body mass index (BMI; categorized as <18.5, 18.5–25, and ≥25 kg/m^2^), and the presence of conditions such as hypertension, hyperlipidemia, diabetes, cardiovascular disease (CVD), and serum uric acid levels. Hypertension was defined as either a prior diagnosis, current use of antihypertensive medications, or measured systolic blood pressure ≥ 140 mmHg and/or diastolic blood pressure ≥ 90 mmHg. Hyperlipidemia was determined by self-reported physician diagnosis or the use of lipid-lowering medications. Diabetes mellitus (DM) was defined as a self-reported physician diagnosis, the use of glucose-lowering drugs, or a fasting plasma glucose level > 126 mg/dl. CVD was identified by any of the following conditions: angina pectoris, myocardial infarction, congestive heart failure, coronary artery disease, or stroke. Uric acid levels were categorized into tertiles: <5.6, 5.6–6.6, and ≥6.6 mg/dl. All covariates were defined according to standard NHANES documentation and established validation criteria.

### 2.5 Survey design and weighting

All analyses accounted for the complex survey design of NHANES, incorporating appropriate sampling strata and primary sampling units (PSUs). Dietary analyses used the day 2 dietary recall weight (WTDR2D), ensuring appropriate weighting for participants with two 24-h dietary recalls. This weight was recalculated to represent the combined 2007–2010 NHANES cycles, ensuring nationally representative estimates of dietary antioxidant and flavonoid intake.

### 2.6 Statistical analyses

Weighted descriptive statistics were calculated using the “svy_tableone” function to compare the intake of antioxidants and flavonoids between PCa and non-cancer groups, while accounting for the complex design of the NHANES survey. Means and standard errors were reported for continuous variables, with comparisons made based on PCa status. All dietary antioxidants and flavonoids were analyzed both as continuous variables and as categorical variables (high vs. low intake groups, based on median values). To assess the relationship between dietary antioxidants, flavonoids, and PCa risk, survey-weighted univariable and multivariable logistic regression models were employed, reporting odds ratios (ORs) and 95% confidence intervals (CIs).

For survival analysis among PCa patients, Kaplan–Meier survival curves were created using survey-weighted techniques to assess differences in overall mortality based on nutrient intake levels, with visual comparisons made accordingly. Survival time (permth_exm) was defined as the number of months from the NHANES Mobile Examination Center (MEC) visit to either the date of death or the end of the follow-up period (31 December 2019, based on the latest NHANES Linked Mortality Files). Survey-weighted univariable and multivariable Cox proportional hazards models were applied to examine the relationship between each antioxidant or flavonoid and overall mortality in PCa patients. These models were developed using svycoxph() within the subset of participants diagnosed with PCa. Each dietary variable was categorized into high vs. low intake groups based on its median value. Hazard ratios (HRs), 95% CIs, and *P*-values were obtained using the broom and gtsummary packages, with the results summarized for all nutrients.

### 2.7 Machine learning models

To further assess the predictive ability of dietary antioxidants and flavonoids for classifying PCa, a series of supervised machine learning models were developed using the tidymodels package in R, A Language and Environment for Statistical Computing (version 4.4.2). The dataset was randomly split into a training set (70%) and a test set (30%), ensuring stratification by PCa status. The models tested included logistic regression, RF, support vector machine (SVM), eXtreme Gradient Boosting (XGBoost), multilayer perceptron (MLP), *k*-nearest neighbors (KNN), and light gradient boosting machine (LightGBM). Hyperparameter tuning was conducted for each model using fivefold cross-validation. Performance was evaluated using metrics such as the area under the receiver operating characteristic curve (AUC), accuracy, sensitivity, specificity, precision-recall AUC, and F1-score.

To understand the internal workings of the best-performing model, SHAP values were calculated. This method evaluates the contribution of each predictor to the model’s output, enabling both global feature ranking and local interpretability. The SHAP value visualization in this study was primarily implemented using the shapviz package. Moreover, the model’s discrimination and clinical relevance were visualized using calibration curves and ROC curve analysis. All preprocessing tasks, including variable encoding and normalization, were executed in a reproducible pipeline utilizing the recipes, parsnip, and workflows components of the tidymodels framework.

### 2.8 Software

All data processing and analyses were conducted using R (version 4.4.2) with packages including survey, nhanesR, tidymodels, randomForest, iml, fastshap, and shapviz.

## 3 Results

### 3.1 Baseline characteristics

**FIGURE 1:**
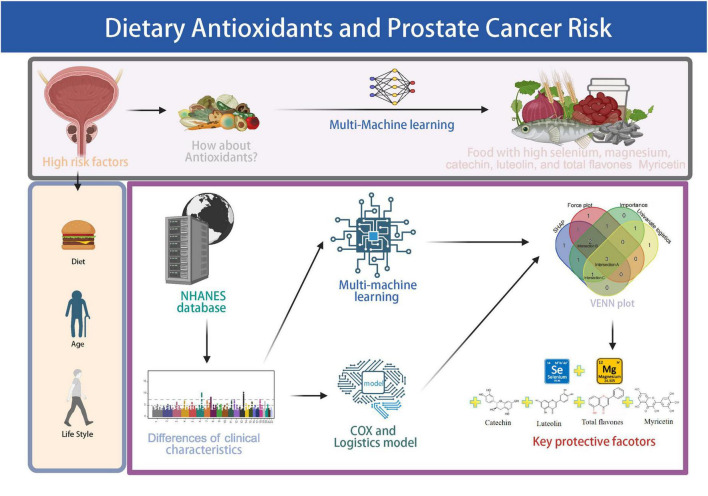
Schematic overview of the study workflow. The pipeline includes NHANES data screening, assessment of antioxidant and flavonoid intake, logistic and Cox regression analyses, machine learning modeling, and identification of key protective dietary factors. Created with BioRender.com.

**FIGURE 2:**
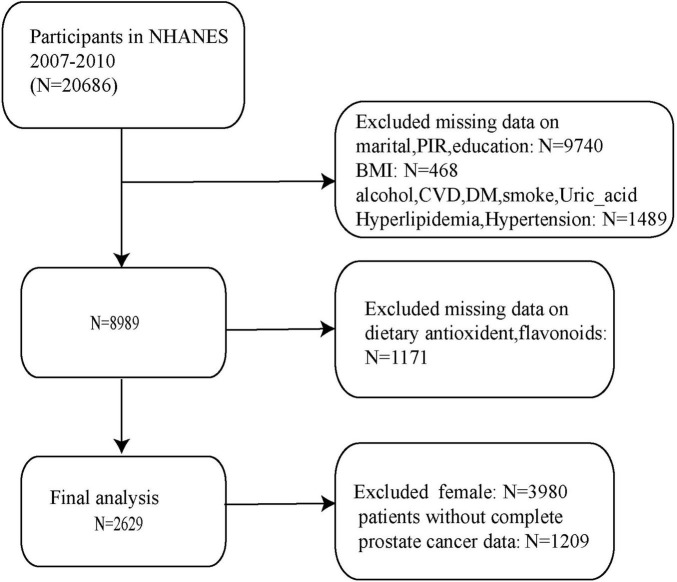
Flow diagram of participant selection from NHANES 2007–2010. Inclusion and exclusion criteria are presented stepwise, leading to the final analytic sample of 2,629 male participants.

To provide an overview of the study design and analytical workflow, a schematic diagram is presented in Figure 1. A total of 2,629 male participants were included in the final analysis after excluding individuals with missing sociodemographic, clinical, or dietary data from the initial NHANES 2007–2010 cohort of 20,686 participants. Of these, 144 participants (5.5%) reported a physician diagnosis of PCa (Figure 2).

Table 1 presents the weighted baseline characteristics and dietary antioxidant comparisons. Participants with PCa were significantly older and had lower intake of selenium, magnesium, quercetin, kaempferol, eriodictyol, epicatechin, and epigallocatechin (all *P* < 0.05). Additionally, lower levels of total flavones and total flavonoids were observed in the PCa group (*P* < 0.05). In addition, higher prevalence of hypertension, CVD, heavy drinker, and former smoking status was observed among PCa cases compared to non-cancer participants (*P* < 0.05). No significant differences were observed in BMI categories, marital status, hyperlipidemia, educational attainment, PIR, diabetes mellitus, or serum uric acid levels between the two groups.

**TABLE 1:** Weighted baseline characteristics and dietary antioxidants and flavonoids intake of participants by prostate cancer status (NHANES 2007–2010).

Variable (mean ± SE)	Total	Pca – no	Pca – yes	*P-*value
Age	56.664 (0.297)	56.160 (0.296)	70.430 (1.526)	<0.001
Magnesium (mg)	336.012 (4.197)	337.036 (4.163)	308.031 (10.267)	0.002
Epicatechin (mg)	11.306 (0.474)	11.387 (0.486)	9.084 (0.882)	0.02
Selenium (mg)	128.074 (1.602)	128.655 (1.626)	112.209 (4.704)	0.001
Epigallocatechin (mg)	19.096 (1.461)	19.392 (1.515)	11.002 (2.635)	0.009
Theaflavin (mg)	1.912 (0.162)	1.943 (0.168)	1.057 (0.287)	0.011
Eriodictyol (mg)	0.181 (0.018)	0.184 (0.018)	0.108 (0.025)	0.012
Luteolin (mg)	0.795 (0.044)	0.801 (0.044)	0.626 (0.057)	0.003
Kaempferol (mg)	5.514 (0.212)	5.581 (0.219)	3.697 (0.476)	<0.001
Quercetin (mg)	13.616 (0.472)	13.736 (0.481)	10.330 (0.977)	0.002
Total_flavones (mg)	1.120 (0.119)	1.131 (0.123)	0.817 (0.073)	0.025
Total_flavonoids (mg)	256.526 (15.589)	259.562 (16.144)	173.545 (28.846)	0.013
**BMI (kg/m^2^), n (%)**				
BMI < 18.5	25 (0.436)	24 (0.436)	1 (0.411)	0.945
18.5 < BMI < 25	547 (19.692)	520 (19.722)	27 (18.874)	
BMI ≥ 25	2,057 (79.873)	1,941 (79.842)	116 (80.715)	
**CVD, n (%)**				
No	2,104 (84.686)	2,010 (85.355)	94 (66.397)	<0.001
Yes	525 (15.314)	475 (14.645)	50 (33.603)	
**Race, n (%)**				
Non-Hispanic White and non-Hispanic Black	1,933 (86.232)	1,804 (85.957)	129 (93.753)	0.047
Mexican American	395 (6.237)	389 (6.389)	6 (2.079)	
Other Hispanic	225 (3.562)	220 (3.650)	5 (1.143)	
Other races – including multi-racial	76 (3.969)	72 (4.003)	4 (3.024)	
**Marital status, n (%)**				
Married/living with partner	1,935 (76.077)	1,826 (75.859)	109 (82.041)	0.194
Live alone	694 (23.923)	659 (24.141)	35 (17.959)	
**Poverty income ratio (PIR), n (%)**				
<1.3	654 (14.918)	626 (14.978)	28 (13.294)	0.611
1.3–3.5	998 (32.641)	931 (32.425)	67 (38.538)	
≥3.5	977 (52.441)	928 (52.597)	49 (48.169)	
**Education level, n (%)**				
≥High school	1,870 (82.571)	1,772 (82.765)	98 (77.268)	0.212
<High school	759 (17.429)	713 (17.235)	46 (22.732)	
**Hyperlipidemia, n (%)**				
Yes	2,105 (81.504)	1,981 (81.353)	124 (85.648)	0.397
No	524 (18.496)	504 (18.647)	20 (14.352)	
**Hypertension, n (%)**				
Yes	1,411 (47.894)	1,308 (47.311)	103 (63.821)	0.049
No	1,218 (52.106)	1,177 (52.689)	41 (36.179)	
**Smoking status, n (%)**				
Former	1,046 (37.764)	970 (37.148)	76 (54.594)	<0.001
Never	1,057 (43.979)	997 (44.098)	60 (40.728)	
Now	526 (18.257)	518 (18.754)	8 (4.678)	
**Diabetes, n (%)**				
Yes	510 (15.628)	35 (16.339)	510 (15.628)	0.797
No	1,975 (84.372)	109 (83.661)	1,975 (84.372)	
**Uric_acid (mg/dl), n (%)**				
<5.6	896 (31.293)	848 (31.080)	48 (37.129)	0.500
≥6.6	914 (35.534)	863 (35.547)	51 (35.179)	
5.5–6.6	819 (33.173)	774 (33.373)	45 (27.692)	
**Alcohol consumption, n (%)**				
Non-drinker or light drinker	1,909 (71.428)	1,786 (70.938)	123 (84.810)	0.037
Heavy drinker	720 (28.572)	699 (29.062)	21 (15.190)	

Continuous variables are presented as weighted means ± SE, and categorical variables as weighted counts and percentages. Comparisons were conducted using survey-weighted linear regression for continuous variables and Rao–Scott Chi-square tests for categorical variables. Weights were applied to account for the complex sampling design of NHANES.

### 3.2 Associations between antioxidants and prostate cancer risk and mortality

Univariable logistic regression analysis demonstrated several significant associations between dietary intake and PCa risk (Supplementary Figure 1). Participants with higher intake levels of magnesium (OR = 0.527, 95% CI: 0.323–0.861, *P* = 0.010), kaempferol (OR = 0.443, 95% CI: 0.251–0.781, *P* = 0.005, based on high vs. low intake group), myricetin (OR = 0.275, 95% CI: 0.142–0.533, *P* < 0.001), and epigallocatechin (OR = 0.443, 95% CI: 0.251–0.781, *P* = 0.020) had a significantly reduced likelihood of PCa compared to those with lower intake. For continuous variables, higher selenium intake (per unit increase) was inversely associated with PCa risk (OR = 0.991, 95% CI: 0.983–0.999, *P* = 0.030), and higher intake of kaempferol also showed a protective association (OR = 0.878, 95% CI: 0.797–0.966, *P* = 0.008). Conversely, higher hesperetin intake was positively associated with PCa risk (OR = 1.010, 95% CI: 1.001–1.019, *P* = 0.036). After adjusting for BMI, marital status, PIR, education, smoking habits, alcohol consumption, diabetes, hypertension, hyperlipidemia, CVD history, race/ethnicity, and other covariates, multivariable logistic regression analysis identified selenium intake (high vs. low, OR = 0.500, 95% CI: 0.329–0.761, *P* = 0.003) as the only dietary factor that remained significantly and independently associated with a lower risk of PCa (Supplementary Table 1).

Univariate Cox regression analyses (Supplementary Figure 2) further supported these associations, showing that lower intake of selenium (HR = 0.993, 95% CI: 0.988–0.998, *P* = 0.005), magnesium (HR = 0.998, 95% CI: 0.997–1.000, *P* = 0.010), kaempferol (HR = 0.927, 95% CI: 0.871–0.987, *P* = 0.019), quercetin (HR = 0.966, 95% CI: 0.938–0.996, *P* = 0.026), luteolin (HR = 0.822, 95% CI: 0.688–0.982, *P* = 0.032), and total flavones (HR = 0.831, 95% CI: 0.697–0.991, *P* = 0.040) was significantly associated with increased mortality risk.

Multivariable Cox regression analysis indicated that higher selenium intake was independently associated with improved overall survival in PCa patients, after adjusting for multiple covariates including BMI, marital status, PIR, CVD, race/ethnicity, education, hyperlipidemia, hypertension, alcohol use, smoking, diabetes, and uric acid levels. Compared to those with lower selenium intake, patients in the higher intake group exhibited a 31.0% lower risk of mortality (HR = 0.690, 95% CI: 0.543–0.877, *P* = 0.002; Supplementary Table 2).

### 3.3 Machine learning performance and feature importance

**FIGURE 3:**
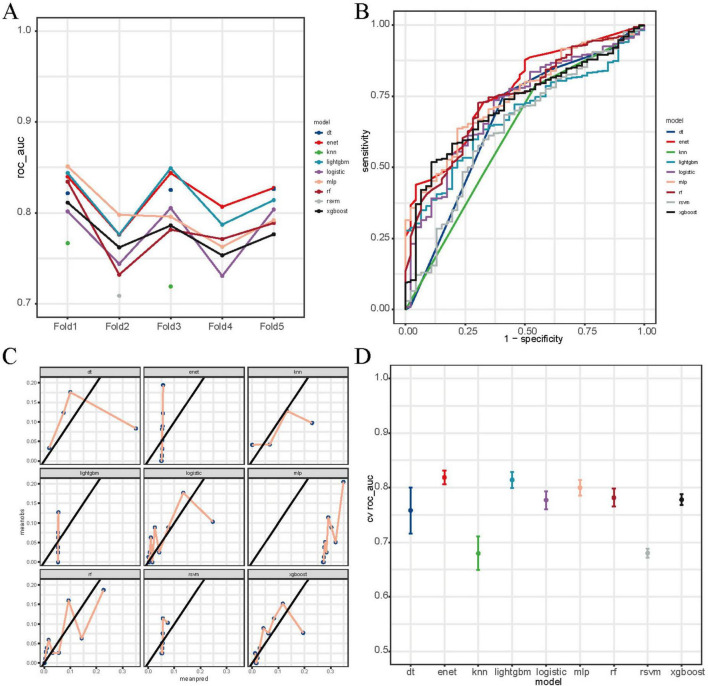
Comparative performance of machine learning models for prostate cancer prediction. **(A)** Fivefold cross-validation ROC AUC scores across nine models: decision tree (dt), elastic net (enet), *k*-nearest neighbors (knn), LightGBM, logistic regression, multilayer perceptron (mlp), random forest (rf), radial SVM (rsvm), and XGBoost. **(B)** Test-set ROC curves showing model discrimination. **(C)** Calibration curves comparing predicted vs. observed probabilities. **(D)** Cross-validated mean ROC AUC scores with standard errors. RF, XGBoost, and MLP showed relatively higher performance.

**FIGURE 4:**
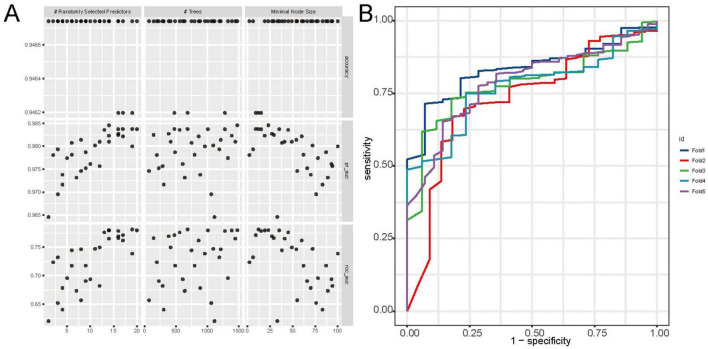
Tuning and cross-validation performance of the random forest (RF) model. **(A)** Grid search results showing the impact of hyperparameters (number of trees, mtry, and node size) on accuracy, pr_auc, and ROC AUC. **(B)** ROC curves from fivefold cross-validation, indicating high and consistent discriminative performance across folds.

Among the nine machine learning models evaluated, the RF algorithm demonstrated high overall predictive performance. As illustrated in Figure 3, RF achieved one of the highest and most stable cross-validated AUC values across all five folds (Figure 3A), and exhibited favorable discriminative ability in ROC curve analysis on the test set (Figure 3B). The calibration plot (Figure 3C) confirmed acceptable calibration of RF predictions. Furthermore, cross-validated ROC AUC scores with standard errors (Figure 3D) indicate that RF, along with elastic net and MLP, maintained robust classification performance. According to Supplementary Table 3, RF achieved the highest accuracy (0.888), sensitivity (0.925), and F1 score (0.940), and a robust ROC AUC of 0.740. While elastic net showed a slightly higher AUC (0.768). Figure 4 provides detailed visualization of the RF model tuning and cross-validation performance. As shown in Figure 4A, the hyperparameter tuning was conducted using a Latin hypercube sampling strategy, which generated 30 combinations of hyperparameters within predefined ranges (number of trees: 50–1500; minimal node size: 1–100; and mtry: 1–20). The optimal configuration identified achieved the best trade-off among classification accuracy, precision-recall AUC, and ROC AUC. Figure 4B illustrates the ROC curves from fivefold cross-validation, indicating stable and consistently high sensitivity and specificity across all folds. Taken together, these findings validate the RF model as the most reliable and generalizable classifier for predicting PCa risk in this study, supported by its superior discriminative power, calibration performance, and validation metrics.

**FIGURE 5:**
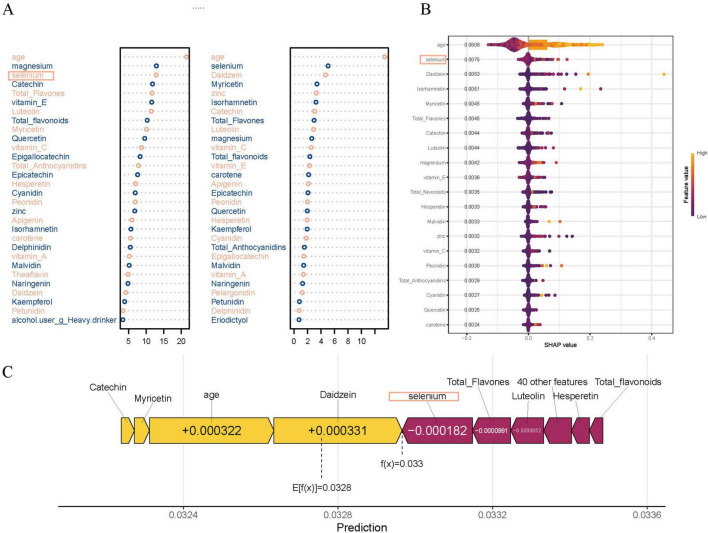
Variable importance in the random forest (RF) model. **(A)** Top dietary and clinical predictors ranked by Mean Decrease Accuracy (MDA) and Mean Decrease Gini (MDG), highlighting the contributions of magnesium, selenium, quercetin, catechin, myricetin, luteolin, and total flavones to model performance. **(B)** SHAP summary plot showing each variable’s marginal contribution and direction of impact on prostate cancer prediction, with color indicating feature value (purple for low and yellow for high). **(C)** SHAP force plot for prediction, illustrating how specific dietary factors such as selenium, catechin, and luteolin decrease prostate cancer risk (shown in purple), while age and Daidzein increase the risk (shown in yellow).

In addition, variable importance analyses in the RF model further emphasized the relevance of specific dietary antioxidants and flavonoids. As shown in Figure 5A, traditional tree-based metrics, including Mean Decrease Accuracy and Mean Decrease Gini, ranked magnesium, selenium, catechin, and total flavonoids among the top contributors to model performance. Figure 5B presents the SHAP summary plot, which quantifies the marginal contribution of each variable to individual predictions. To further illustrate the prediction mechanism, a force plot (Figure 5C) was generated. In this visualization, dietary factors such as selenium, luteolin, and total flavones were highlighted in purple, indicating their negative contribution to the predicted PCa risk, while age and Daidzein were shown in yellow, representing positive contributions to the risk estimate.

**FIGURE 6:**
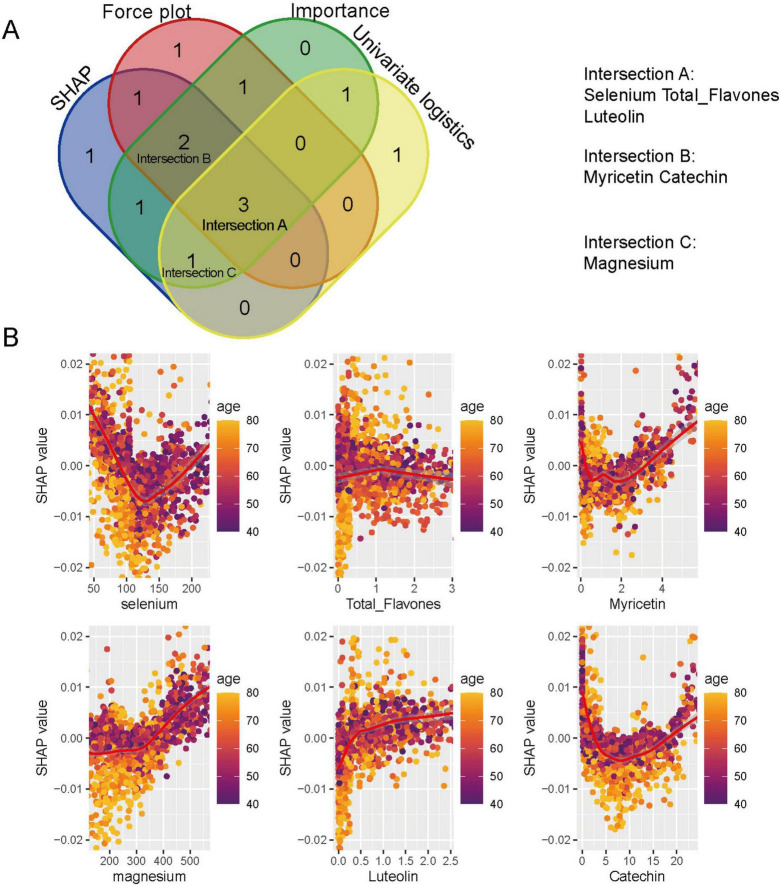
Key dietary predictors identified by random forest and SHAP analyses. **(A)** Venn diagram showing the overlap of top predictors identified by SHAP values, Mean Decrease Accuracy (MDA), univariate logistic regression, and force plot analyses. Selenium, total flavones, luteolin (Intersection A); myricetin, catechin (Intersection B); and magnesium (Intersection C) were identified. **(B)** SHAP scatter plots illustrating the non-linear associations between dietary antioxidants and prostate cancer risk, stratified by age.

By integrating the top dietary predictors identified through SHAP values, force plot contributions, variable importance rankings, and univariate logistic regression analyses, three sets of overlapping key features were identified (Figure 6A). Specifically, selenium, total flavones, and luteolin were selected by all four methods (Intersection A); myricetin and catechin were identified by three methods (Intersection B); and magnesium was highlighted by SHAP, importance, and univariate logistics analyses (Intersection C). Correlation analyses of SHAP values with intake levels and age (Figure 6B) demonstrated distinct distribution patterns for each feature. Selenium, myricetin, and catechin showed U-shaped distributions, while magnesium and luteolin displayed monotonic changes. Total flavones exhibited variation with age, though no clear trend was observed in the overall population.

Survival analysis using Kaplan–Meier curves demonstrated that lower intake of selenium, magnesium, myricetin, luteolin, and total flavones was significantly associated with reduced overall survival among PCa patients (Supplementary Figure 3). No significant association was observed for catechin intake.

## 4 Discussion

This study examined the association of dietary antioxidant and flavonoid intake with PCa risk and survival using NHANES 2007–2010 data. Key dietary components, including selenium, magnesium, myricetin, catechin, luteolin, and total flavones, were identified as potential factors influencing PCa risk and survival outcomes. SHAP-based analyses revealed complex, non-linear associations, including U-shaped patterns for selenium, myricetin, and catechin, suggesting that both low and excessive intakes may elevate PCa risk, while moderate intake offers potential protective benefits. Magnesium and luteolin demonstrated dose-dependent relationships, with protective effects observed up to a threshold, beyond which higher intake was associated with increased risk. Interestingly, total flavones did not exhibit a clear SHAP pattern but showed a protective trend in older individuals, indicating potential age-specific effects. These associations were supported by both traditional regression analyses and RF model, which demonstrated strong predictive performance and interpretability.

Selenium and magnesium have long been investigated for their potential anticancer properties. Evidence from observational studies and meta-analyses suggests that selenium status is inversely associated with PCa risk (13, 14). Mechanistically, selenium exerts its anticancer effects through multiple, dose-dependent biological pathways. At nutritional levels, selenium is incorporated into essential selenoproteins, such as glutathione peroxidases (e.g., GPx4) and thioredoxin reductases, which maintain redox homeostasis and protect cells against oxidative damage (15). These antioxidant activities contribute to DNA protection, regulation of cellular redox balance, and modulation of immune responses. However, at supranutritional levels, selenium compounds, particularly in the form of selenite or methylselenol metabolites, can exert prooxidant effects by promoting the generation of ROS. This oxidative stress can lead to DNA strand breaks, activation of p53, and induction of apoptosis via mitochondrial pathways (16). Selenium has also been shown to modulate critical signaling cascades, including NF-κB, PI3K/Akt, and MAPK pathways (16–18), which are involved in cell proliferation, survival, and inflammation. Moreover, selenium compounds may trigger various forms of cell death beyond apoptosis, such as ferroptosis, necroptosis, and autophagy, expanding their potential in targeting resistant tumor phenotypes (16). Interestingly, selenium may exert synergistic effects when combined with other bioactive compounds, such as silybin, leading to a reduction in prostate-specific antigen (PSA) levels and modulation of growth-related pathways in PCa cells (19, 20). Magnesium, an essential mineral for numerous enzymatic reactions, plays a pivotal role in maintaining genomic stability, regulating apoptosis, and modulating immune responses processes critically involved in carcinogenesis (21, 22). Recent analyses of NHANES 2005–2018 data demonstrated a significant association between higher magnesium depletion scores (MDS) and increased PCa risk, with individuals exhibiting an MDS ≥ 3 having over a threefold increased risk compared to those with MDS = 0 (22). Mechanistically, magnesium deficiency promotes tumorigenesis by exacerbating oxidative stress, chronic inflammation, and DNA damage, thereby fostering genetic instability (23). Magnesium also regulates intracellular calcium homeostasis; its deficiency may lead to calcium dysregulation, a hallmark of several cancers (22). Furthermore, low magnesium levels have been shown to enhance epithelial-mesenchymal transition (EMT), facilitating cellular plasticity, migration, and metastasis. Beyond these effects, magnesium supports the function of cytotoxic T cells, suggesting a potential role in modulating the tumor immune microenvironment (24). These findings underscore the complex and context-dependent roles of magnesium in cancer biology and highlight its potential as both a biomarker and a therapeutic target for prostate and other malignancies.

Flavonoids, particularly luteolin, myricetin, catechin, and total flavones, were inversely associated with PCa risk in this study. Mechanistically, flavonoids exert multifaceted anticancer effects by modulating several biological pathways relevant to prostate carcinogenesis. These polyphenolic compounds act as potent antioxidants, mitigating oxidative DNA damage, and suppress chronic inflammation, which is a key driver of tumor initiation and progression (25–27). In PCa cells, flavonoids such as luteolin and myricetin have been shown to inhibit cell proliferation and induce apoptosis by modulating the expression of pro- and anti-apoptotic proteins, as well as by disrupting mitochondrial membrane potential (28, 29). Furthermore, they suppress AR signaling, a critical pathway in PCa development, and downregulate oncogenic signaling cascades including PI3K/Akt, MAPK, and NF-κB (25). These actions collectively contribute to cell cycle arrest, reduced metastasis, and inhibition of EMT (30). Notably, emerging evidence also suggests flavonoids may enhance immune surveillance and modulate the tumor microenvironment, reinforcing their potential as dietary components in cancer prevention (26). These findings highlight the multifaceted mechanisms through which flavonoids may exert protective effects against PCa, including antioxidative, anti-inflammatory, pro-apoptotic, and anti-androgenic activities.

In the machine learning analysis, particularly the RF model, provided strong validation of the associations identified through traditional weighted regression. Unlike traditional regression methods that assume linearity and limited interactions, machine learning algorithms can flexibly capture complex, non-linear relationships among high-dimensional dietary features. In this study, nine supervised classification models were evaluated, including logistic regression, elastic net, decision tree, RF, SVM, KNN, MLP, XGBoost, and LightGBM. Each model offers unique advantages in terms of performance, interpretability, or computational efficiency. Among these, the RF model demonstrated the best overall predictive accuracy and robustness. RF is particularly well-suited for clinical datasets characterized by a large number of variables and potential non-linear interactions. It builds an ensemble of decision trees through bootstrap aggregation and random feature selection, enabling the model to automatically identify complex patterns without assuming predefined relationships. This architecture reduces the risk of overfitting compared to single-tree models and yields an unbiased estimation of model performance via out-of-bag (OOB) error, thus reducing reliance on external validation datasets. Additionally, RF allows for intuitive variable importance ranking and integration with SHAP values to quantify feature contributions (11). To enhance the interpretability of the machine learning models, we employed multiple complementary approaches for feature importance assessment. First, traditional tree-based metrics (e.g., Mean Decrease Accuracy and Mean Decrease Gini) ranked variables based on their contribution to model accuracy but may bias toward features with higher variability. Second, SHAP summary plots provided a global view of how each feature influenced the model’s predictions across the entire dataset, allowing for visualization of both directionality and effect size. Third, SHAP force plots highlighting the contribution of individual features to the predicted risk for each participant. While traditional metrics offer a straightforward ranking, SHAP values provide nuanced, interpretable insights into complex, non-linear relationships but are computationally intensive and sensitive to collinearity. Importantly, by integrating findings from machine learning models with results from univariable logistic regression analyses, we were able to identify consistent and robust dietary predictors, such as selenium, magnesium, catechin, myricetin, luteolin, and total flavone across multiple analytical frameworks. This triangulation of evidence strengthens the reliability of the identified associations.

In comparison with previous studies (31, 32), our findings offer new insights by integrating survey-weighted regression with interpretable machine learning. Our findings revealed complex, non-linear, and age-specific interactions between these factors and PCa outcomes. Through SHAP analyses, we observed U-shaped associations for selenium, myricetin, and catechin, suggesting that both insufficient and excessive intakes may increase PCa risk, while moderate intake confers protection. Selenium’s protective range was particularly evident at intermediate intake levels, with stronger protective effects observed in older participants. This may explain why the SELECT trial, which focused on selenium supplementation, did not demonstrate a clear protective effect (33). In light of our findings, dietary strategies for PCa prevention may benefit from emphasizing foods rich in selenium, magnesium, catechin, myricetin, luteolin, and total flavones, which is consistent with the majority of previous studies (3, 5, 13, 29, 34). Some studies have also reported that excessive intake of certain flavonoids may be positively associated with PCa risk, indicating potential dose-dependent effects (35, 36). For example, Wang et al. (35) observed a positive association between total flavonoid intake and PCa risk at the highest intake quintile in a prospective U.S. cohort study. Similarly, Reale et al. (36) reported that high flavanone intake was linked to increased PCa risk, although flavonols and catechins were associated with a lower risk. These findings suggest that while moderate flavonoid consumption may be protective, excessive intake could potentially be detrimental. However, due to the limited number of high-intake individuals and the lack of robust clinical trials, these dose-response relationships remain uncertain and warrant further investigation. From a nutritional standpoint, many of these compounds are abundant in commonly consumed plant-based foods. Magnesium is prevalent in leafy green vegetables, whole grains, legumes, and nuts; selenium in Brazil nuts, seafood, and organ meats; catechins in green and black tea, cocoa, and certain fruits such as apples and berries; myricetin in onions, grapes, berries, and red wine; luteolin in celery, parsley, thyme, green peppers, and chamomile tea; and total flavones in a wide variety of fruits and vegetables. These dietary sources align with evidence-based guidelines from the World Cancer Research Fund and the American Institute for Cancer Research, which recommend a plant-forward diet low in red meat, dairy products, and saturated fats for cancer prevention (37, 38). Overall, these findings underscore the need for personalized dietary strategies that account for individual risk profiles, age, and intake levels when considering flavonoid supplementation or dietary modifications for PCa prevention and management.

Despite the strengths of this study, several limitations must be considered. First, the cross-sectional design of NHANES limits the ability to infer causality; for example, individuals diagnosed with PCa may have modified their dietary habits as a result of the disease. Second, dietary intake was measured using two 24-h recall, which may not accurately capture long-term dietary patterns. Third, PCa diagnosis relied on self-reporting, which introduces the potential for recall bias. Moreover, despite controlling for various covariates, residual confounding remains a possibility. Furthermore, while the application of NHANES survey weights helps mitigate some biases, the relatively small number of PCa cases and the exclusion of a substantial number of participants may have introduced selection bias, potentially impacting the generalizability of the findings. Additionally, the relatively low ROC AUC value of the RF model (0.740) may be partly attributed to the absence of key variables, such as genetic risk factors, family history of PCa, medication use (e.g., statins and NSAIDs), and hormonal status, which were not available in the NHANES database. Future research should aim to incorporate a broader range of factors, such as genetic background, to improve risk prediction and ensure more comprehensive insights into PCa prevention and management. However, the study benefits from the use of nine supervised machine learning models, enabling a thorough comparison and validation of predictive accuracy. The use of SHAP-based interpretation further enhances the transparency of the models and aids in identifying significant dietary factors associated with PCa (12).

## 5 Conclusion

In this nationally representative U.S. cohort, higher intake of selenium, magnesium, catechin, myricetin, luteolin, and total flavones was associated with a lower risk of PCa, and lower intake of several flavonoids predicted poorer survival. Machine learning and regression analyses consistently supported these findings, revealing complex dose-response and age-related patterns. These results suggest that antioxidant- and flavonoid-rich diets may contribute to PCa prevention and improved outcomes. Future longitudinal and interventional studies are essential to confirm causality and refine dietary recommendations.

## Data Availability

The original contributions presented in this study are included in this article/Supplementary material, further inquiries can be directed to the corresponding authors.

## Footnotes

1
https://www.ars.usda.gov/northeast-area/beltsville-md-bhnrc/beltsville-human-nutrition-research-center/food-surveys-research-group/docs/fndds-flavonoid-database/

## Supplementary material

Supplementary Figure 1 Survey-weighted Forest plot of univariate logistic regression results assessing the association between dietary antioxidant and flavonoid intake and prostate cancer risk. Odds ratios (ORs) and 95% confidence intervals (CIs) are shown for each nutrient.

Supplementary Figure 2 Survey-weighted Forest plot of univariate Cox regression analyses showing adjusted hazard ratios (HRs) and 95% confidence intervals (CIs) for dietary antioxidants and flavonoids in relation to prostate cancer risk. Models adjust for demographic, clinical, and lifestyle covariates.

Supplementary Figure 3 Kaplan–Meier survival curves for prostate cancer patients stratified by dietary intake levels (high vs. low) of selenium, total flavones, myricetin, magnesium, luteolin, and catechin. Survival probabilities were compared using log-rank tests; *P* values are shown in each panel.

Supplementary Table 1 Survey-weighted multivariable logistic regression analysis of selenium and covariates in relation to prostate cancer risk. Survey-weighted multivariable logistic regression analysis evaluating the association between selenium intake (categorized as high vs. low based on median intake) and prostate cancer risk, adjusted for key demographic, lifestyle, and clinical covariates. Odds ratios (ORs), 95% confidence intervals (CIs), and corresponding *P-* values are reported.

Supplementary Table 2 Survey-weighted multivariable cox regression analysis of selenium and covariates in relation to prostate cancer mortality risk. Survey-weighted multivariable Cox regression analysis examining the association between selenium intake and prostate cancer mortality, adjusted for BMI, marital status, poverty income ratio (PIR), cardiovascular disease (CVD), race/ethnicity, education level, hyperlipidemia, hypertension, alcohol consumption, smoking status, diabetes, and uric acid levels. Hazard ratios (HRs), 95% confidence intervals (CIs), and *P* values are presented.

Supplementary Table 3 Performance metrics of nine machine learning models for prostate cancer classification. Performance metrics of nine machine learning models for prostate cancer classification. Indicators include accuracy, sensitivity, specificity, precision, F1-score, ROC AUC, and others, highlighting the predictive strength and diagnostic consistency across models.
