# Supplementary figures and images for: Dietary antioxidants and flavonoids are inversely associated with prostate cancer risk and mortality: evidence from NHANES and machine learning
Source: Front Nutr. 2025 Jul 8;12:1611848. doi: 10.3389/fnut.2025.1611848 (PMC12282170; doi:10.3389/fnut.2025.1611848)

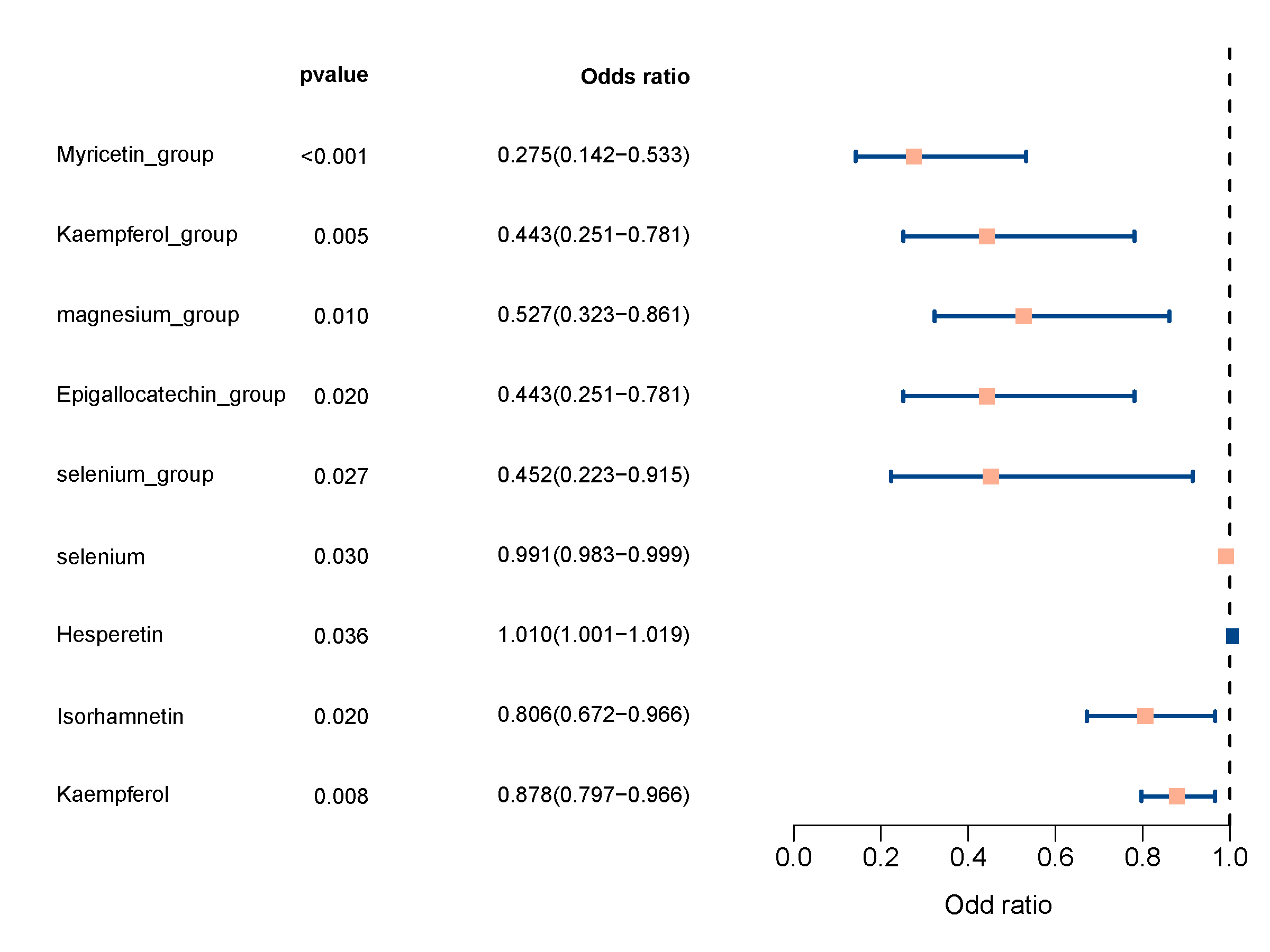

Supplement: Supplementary Figure 1 — Survey-weighted Forest plot of univariate logistic regression results assessing the association between dietary antioxidant and flavonoid intake and prostate cancer risk. Odds ratios (ORs) and 95% confidence intervals (CIs) are shown for each nutrient. [file Image_1.jpeg]

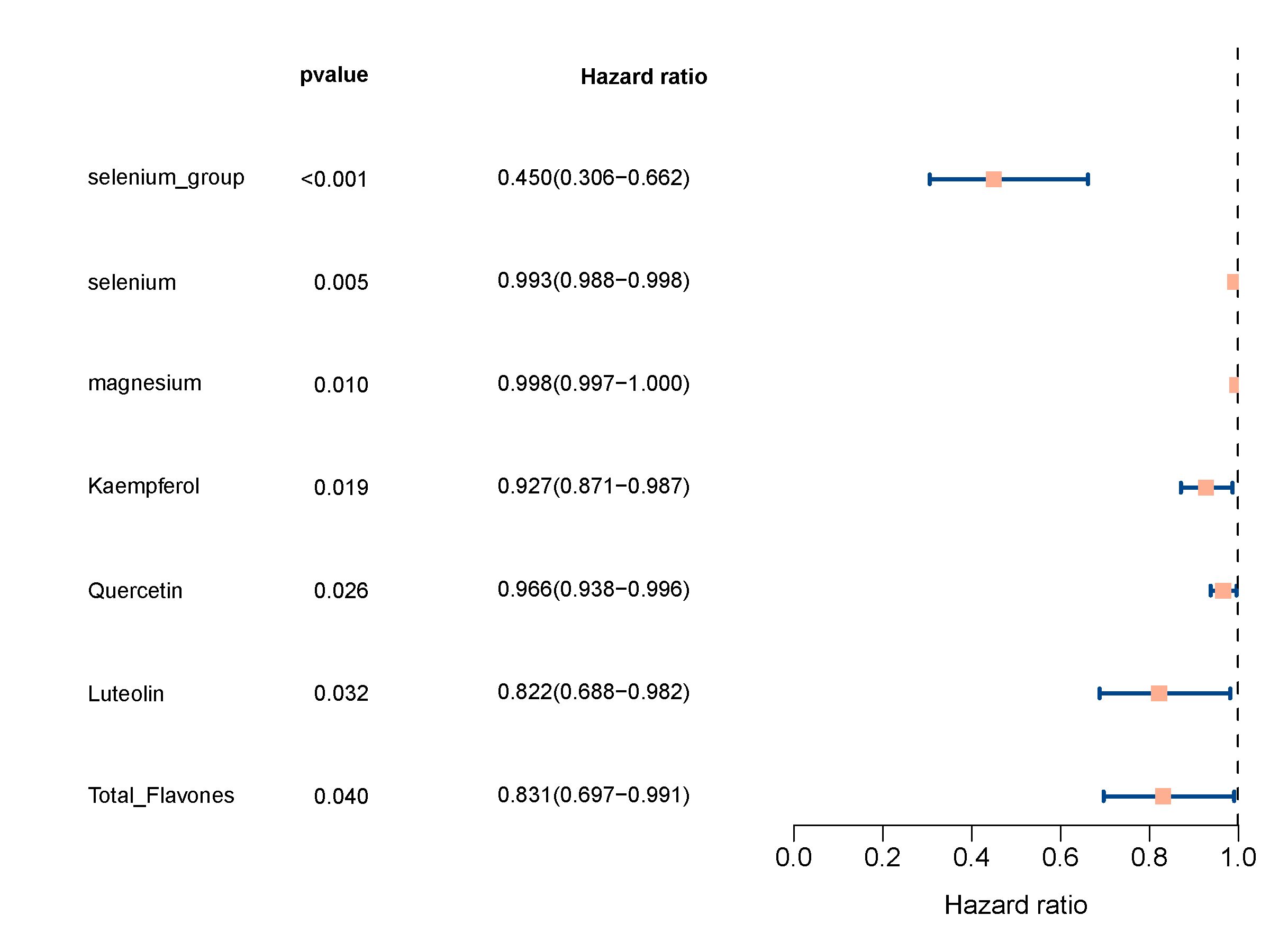

Supplement: Supplementary Figure 2 — Survey-weighted Forest plot of univariate Cox regression analyses showing adjusted hazard ratios (HRs) and 95% confidence intervals (CIs) for dietary antioxidants and flavonoids in relation to prostate cancer risk. Models adjust for demographic, clinical, and lifestyle covariates. [file Image_2.jpeg]

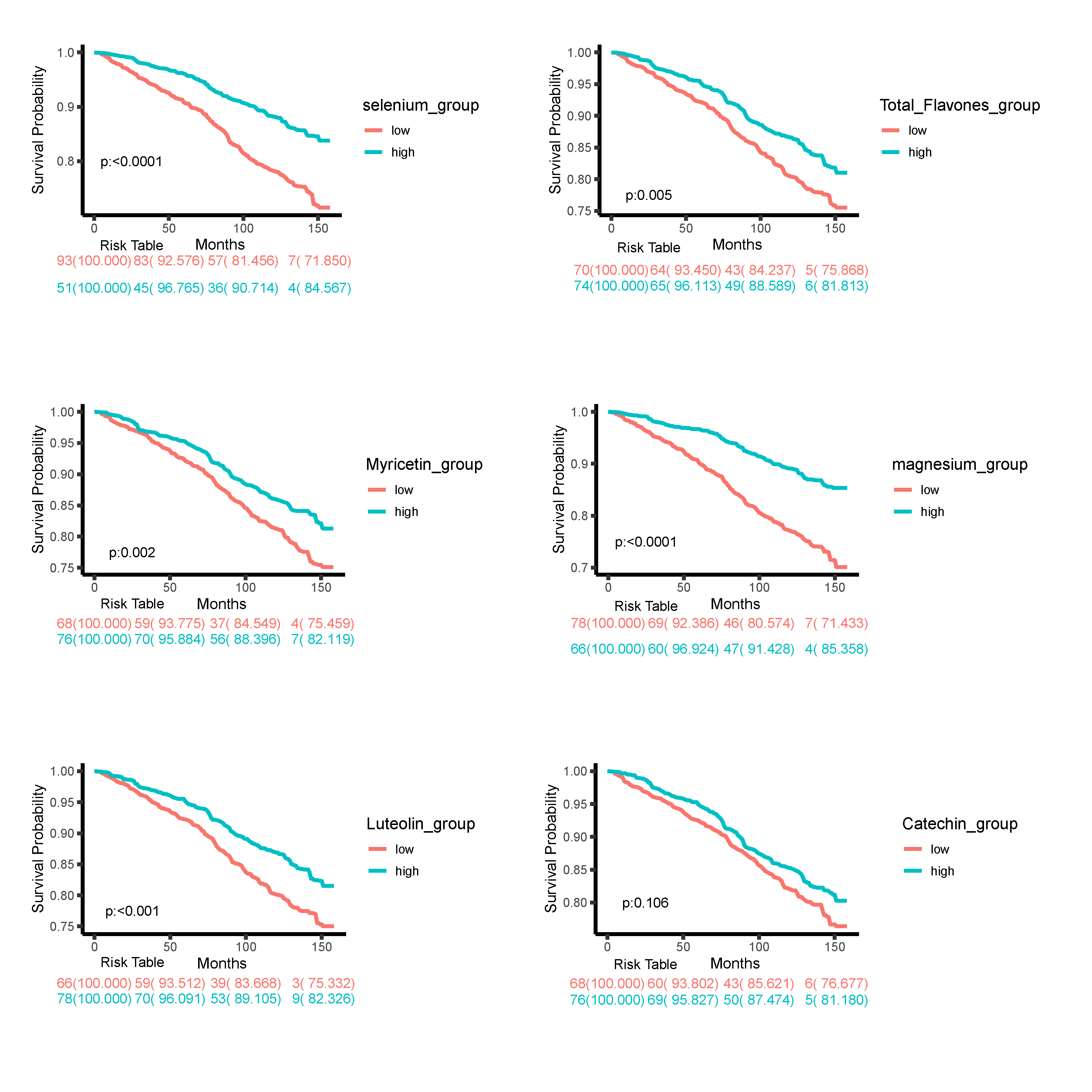

Supplement: Supplementary Figure 3 — Kaplan–Meier survival curves for prostate cancer patients stratified by dietary intake levels (high vs. low) of selenium, total flavones, myricetin, magnesium, luteolin, and catechin. Survival probabilities were compared using log-rank tests; P values are shown in each panel. [file Image_3.jpeg]
