# Supplementary material for: Dietary antioxidants and flavonoids are inversely associated with prostate cancer risk and mortality: evidence from NHANES and machine learning
Source: Front Nutr. 2025 Jul 8;12:1611848. doi: 10.3389/fnut.2025.1611848 (PMC12282170; doi:10.3389/fnut.2025.1611848)
Supplement: Supplementary Table 1 — Survey-weighted multivariable logistic regression analysis of selenium and covariates in relation to prostate cancer risk. Survey-weighted multivariable logistic regression analysis evaluating the association between selenium intake (categorized as high vs. low based on median intake) and prostate cancer risk, adjusted for key demographic, lifestyle, and clinical covariates. Odds ratios (ORs), 95% confidence intervals (CIs), and corresponding P- values are reported. [file Table_1.docx]

**Supplementary table S1. Survey-weighted multivariable logistic regression analysis of selenium and covariates in relation to prostate cancer risk.**

| **Variable** | **OR** | **95% CI** | **P value** |
| --- | --- | --- | --- |
| **selenium_group** | |  |  |
| low | ref | ref | ref |
| high | 0.500 | 0.500(0.329,0.761) | 0.003 |
| **BMI(kg/m^2^)** |  |  |  |
| BMI < 18.5 | ref | ref | ref |
| 18.5 < BMI < 25 | 0.723 | 0.723(0.077,6.824) | 0.760 |
| BMI ≥ 25 | 0.653 | 0.653(0.061,6.993) | 0.704 |
| **Marital status** |  |  |  |
| Married/living with partner | ref | ref | ref |
| Live alone | 0.770 | 0.770(0.430,1.379) | 0.350 |
| **Poverty income ratio (PIR)** |  |  |  |
| <1.3 | ref | ref | ref |
| 1.3–3.5 | 1.045 | 1.045(0.548,1.995) | 0.884 |
| ≥3.5 | 0.947 | 0.947(0.461,1.943) | 0.872 |
| **CVD** |  |  |  |
| No | ref | ref | ref |
| Yes | 2.192 | 2.192(1.157,4.153) | 0.02 |
| **Race** |  |  |  |
| Non-Hispanic White and Non-Hispanic Black | ref | ref | ref |
| Mexican American | 0.276 | 0.276(0.100,0.759) | 0.017 |
| Other Hispanic | 0.277 | 0.277(0.077,0.997) | 0.05 |
| Other races - including Multi-Racial | 0.636 | 0.636(0.151,2.674) | 0.508 |
| **Education level** | |  |  |
| ≥High school | ref | ref | ref |
| <High school | 1.735 | 1.735(0.957,3.147) | 0.067 |
| **Hyperlipidemia** | |  |  |
| Yes | ref | ref | ref |
| No | 0.704 | 0.704(0.304,1.633) | 0.384 |
| **Hypertension** | |  |  |
| Yes | ref | ref | ref |
| No | 0.649 | 0.649(0.297,1.417) | 0.253 |
| **Alcohol consumption** | |  |  |
| Nondrinker or Light drinker | ref | ref | ref |
| Heavy drinker | 0.669 | 0.669(0.299,1.497) | 0.301 |
| **Smoking status** |  |  |  |
| former | ref | ref | ref |
| never | 0.743 | 0.743(0.476,1.159) | 0.173 |
| now | 0.168 | 0.168(0.049,0.574) | 0.008 |
| **Diabetes** |  |  |  |
| Yes | ref | ref | ref |
| No | 1.575 | 1.575(0.961,2.581) | 0.069 |
| **Uric_acid (mg/dl)** | |  |  |
| <5.6 | ref | ref | ref |
| ≥6.6 | 0.719 | 0.719(0.423,1.223) | 0.202 |
| 5.5–6.6 | 0.722 | 0.722(0.327,1.593) | 0.39 |
