# Supplementary material for: Dietary antioxidants and flavonoids are inversely associated with prostate cancer risk and mortality: evidence from NHANES and machine learning
Source: Front Nutr. 2025 Jul 8;12:1611848. doi: 10.3389/fnut.2025.1611848 (PMC12282170; doi:10.3389/fnut.2025.1611848)
Supplement: Supplementary Table 2 — Survey-weighted multivariable cox regression analysis of selenium and covariates in relation to prostate cancer mortality risk. Survey-weighted multivariable Cox regression analysis examining the association between selenium intake and prostate cancer mortality, adjusted for BMI, marital status, poverty income ratio (PIR), cardiovascular disease (CVD), race/ethnicity, education level, hyperlipidemia, hypertension, alcohol consumption, smoking status, diabetes, and uric acid levels. Hazard ratios (HRs), 95% confidence intervals (CIs), and P values are presented. [file Table_2.docx]

**Supplementary table S2. Survey-weighted multivariable cox regression analysis of selenium and covariates in relation to prostate cancer mortality risk.**

| **character** | **HR** | **95% CI** | **P value** |
| --- | --- | --- | --- |
| **selenium_group** | |  |  |
| low | ref | ref | ref |
| high | 0.690 | 0.690(0.543,0.877) | 0.002 |
| **BMI(kg/m^2^)** |  |  |  |
| BMI < 18.5 | ref | ref | ref |
| 18.5 < BMI < 25 | 0.699 | 0.699(0.360,1.359) | 0.291 |
| BMI ≥ 25 | 0.572 | 0.572(0.301,1.088) | 0.089 |
| **Marital status** |  |  |  |
| Married/living with partner | ref | ref | ref |
| Live alone | 1.497 | 1.497(1.180,1.898) | <0.001 |
| **Poverty income ratio (PIR)** |  |  |  |
| <1.3 | ref | ref | ref |
| 1.3–3.5 | 1.085 | 1.085(0.820,1.437) | 0.567 |
| ≥3.5 | 0.484 | 0.484(0.329,0.711) | <0.001 |
| **CVD** |  |  |  |
| No | ref | ref | ref |
| Yes | 2.126 | 2.126(1.659,2.725) | <0.001 |
| **Race** |  |  |  |
| Non-Hispanic White and Non-Hispanic Black | ref | ref | ref |
| Mexican American | 0.410 | 0.410(0.285,0.591) | <0.001 |
| Other Hispanic | 0.727 | 0.727(0.459,1.149) | 0.172 |
| Other races - including Multi-Racial | 0.943 | 0.943(0.411,2.160) | 0.889 |
| **Education level** | |  |  |
| ≥High school | ref | ref | ref |
| <High school | 1.414 | 1.414(1.018,1.963) | 0.039 |
| **Hyperlipidemia** | |  |  |
| No | ref | ref | ref |
| Yes | 1.07 | 1.070(0.750,1.528) | 0.708 |
| **Hypertension** | |  |  |
| No | ref | ref | ref |
| Yes | 0.671 | 0.671(0.504,0.893) | 0.006 |
| **Alcohol** | |  |  |
| Nondrinker or Light drinker | ref | ref | ref |
| Heavy drinker | 0.580 | 0.580(0.399,0.842) | 0.004 |
| **Smoking status** |  |  |  |
| former | ref | ref | ref |
| never | 0.591 | 0.591(0.440,0.794) | <0.001 |
| now | 0.836 | 0.836(0.576,1.213) | 0.346 |
| **Diabetes** |  |  |  |
| Yes | ref | ref | ref |
| No | 0.876 | 0.876(0.699,1.097) | 0.249 |
| **Uric_acid(mg/dl)** | |  |  |
| <5.6 | ref | ref | ref |
| ≥6.6 | 0.972 | 0.972(0.773,1.222) | 0.807 |
| 5.5–6.6 | 0.784 | 0.784(0.594,1.034) | 0.085 |
