# Supplementary material for: Dietary antioxidants and flavonoids are inversely associated with prostate cancer risk and mortality: evidence from NHANES and machine learning
Source: Front Nutr. 2025 Jul 8;12:1611848. doi: 10.3389/fnut.2025.1611848 (PMC12282170; doi:10.3389/fnut.2025.1611848)
Supplement: Supplementary Table 3 — Performance metrics of nine machine learning models for prostate cancer classification. Performance metrics of nine machine learning models for prostate cancer classification. Indicators include accuracy, sensitivity, specificity, precision, F1-score, ROC AUC, and others, highlighting the predictive strength and diagnostic consistency across models. [file Table_3.docx]

| **Model** | **Accuracy** | **Kappa** | **Sensitivity** | **Specificity** | | **PPV** | **NPV** | **MCC** | **Youden Index** | **Balanced Accuracy** | **Detection Prevalence** | **Precision** | **Recall** | **F1 Score** | **ROC AUC** |
| --- | --- | --- | --- | --- | --- | --- | --- | --- | --- | --- | --- | --- | --- | --- | --- |
| Logistic | 0.664 | 0.094 | 0.665 | 0.652 | 0.969 | | 0.108 | 0.155 | 0.317 | 0.659 | 0.646 | 0.969 | 0.665 | 0.789 | 0.716 |
| Elastic Net | 0.697 | 0.125 | 0.697 | 0.696 | 0.974 | | 0.125 | 0.196 | 0.393 | 0.696 | 0.674 | 0.974 | 0.697 | 0.813 | 0.768 |
| Decision Tree | 0.748 | 0.130 | 0.758 | 0.587 | 0.967 | | 0.130 | 0.184 | 0.345 | 0.672 | 0.738 | 0.967 | 0.758 | 0.850 | 0.663 |
| **Random Forest** | 0.888 | 0.184 | 0.925 | 0.304 | 0.955 | | 0.200 | 0.189 | 0.229 | 0.614 | 0.911 | 0.955 | 0.925 | 0.940 | 0.740 |
| XGBoost | 0.816 | 0.094 | 0.847 | 0.326 | 0.953 | | 0.116 | 0.109 | 0.173 | 0.586 | 0.837 | 0.953 | 0.847 | 0.897 | 0.718 |
| rSVM | 0.760 | 0.066 | 0.785 | 0.370 | 0.953 | | 0.096 | 0.087 | 0.154 | 0.577 | 0.776 | 0.953 | 0.785 | 0.861 | 0.639 |
| MLP | 0.720 | 0.110 | 0.728 | 0.587 | 0.966 | | 0.118 | 0.163 | 0.315 | 0.658 | 0.710 | 0.966 | 0.728 | 0.830 | 0.749 |
| LightGBM | 0.777 | 0.069 | 0.803 | 0.348 | 0.952 | | 0.099 | 0.088 | 0.151 | 0.576 | 0.795 | 0.952 | 0.803 | 0.872 | 0.678 |
| KNN | 0.768 | 0.104 | 0.787 | 0.457 | 0.959 | | 0.117 | 0.136 | 0.244 | 0.622 | 0.773 | 0.959 | 0.787 | 0.865 | 0.620 |

**Supplementary Table S3.** Performance metrics of nine machine learning models for prostate cancer classification.
